# Vancomycin-Associated Leukocytoclastic Vasculitis

**DOI:** 10.1155/2011/356370

**Published:** 2011-06-30

**Authors:** Makhawadee Pongruangporn, David J. Ritchie, Dongsi Lu, Jonas Marschall

**Affiliations:** ^1^Division of Infectious Diseases, Washington University School of Medicine, St. Louis, MO 63110, USA; ^2^Pharmacy Practice Division, Department of Pharmacy and St. Louis College of Pharmacy, Barnes-Jewish Hospital, MO 63110, USA; ^3^Department of Pathology and Immunology, Washington University School of Medicine, St. Louis, MO 63110, USA

## Abstract

Vancomycin is U.S. Food and Drug Administration (FDA) approved for treatment of serious infections caused by methicillin-resistant Staphylococcus aureus (MRSA) or in individuals who have failed, cannot tolerate, or are allergic to other antibiotics. Very few cases of vancomycin-associated leukocytoclastic vasculitis have been published. We report on a patient who developed pruritus and palpable purpura in both lower extremities after receiving six days of intravenous vancomycin. Skin biopsy revealed leukocytoclastic vasculitis.

## 1. Case Report and Discussion

**Figure 1:**
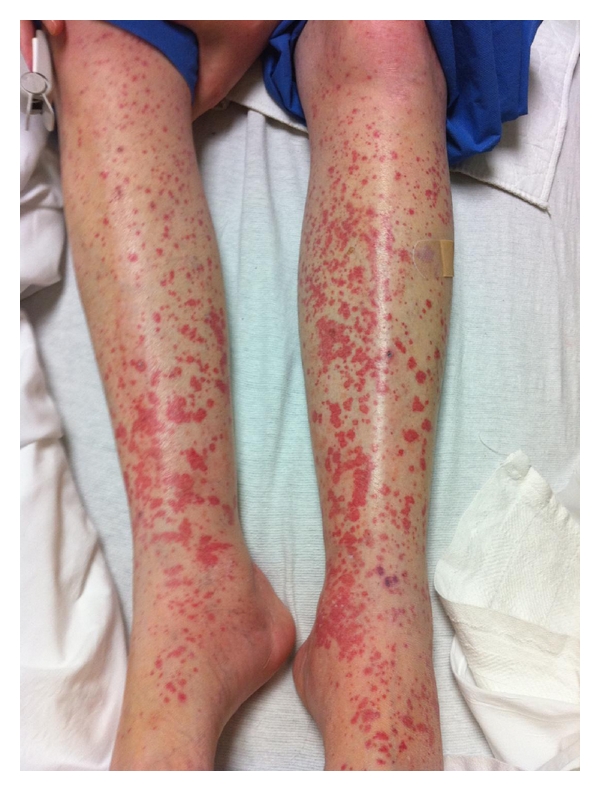
On day 6 after starting vancomycin, the patient develops a pruritic, nonblanching erythematous rash on both legs, buttocks and lower back area.

**Figure 2:**
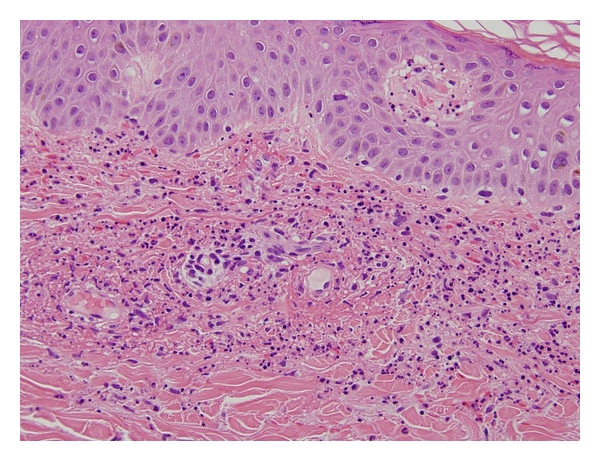
High power view (400x) shows perivascular neutrophilic infiltrate with fibrinoid necrosis of vessel wall, abundant nuclear dust and red blood cell extravasation.

A 52-year-old white female with history of intravenous heroin abuse presented with a 10-day history of productive cough associated with right-sided pleuritic chest pain. She had a past medical history of one generalized tonic-clonic seizure in 2007 for which she was on phenytoin without evidence of further episodes; phenytoin was stopped by the patient three months prior to this admission. Other medications administered during her hospital stay include tizanidine, albuterol/ipratropium inhaler, docusate, clonazepam, lidocaine patch, and heparin sulfate subcutaneous injection. On physical examination she was febrile with maximum temperature of 100.6°F (38.1°C). She had decreased breath sounds over her right lower lung and CXR, and CT chest demonstrated right-sided pleural effusion. Blood cultures and cultures from right thoracentesis specimens revealed MRSA bacteremia and empyema, respectively, for which the patient was started on vancomycin 1 gm IV q 12 hours. This dose provided a vancomycin steady state serum trough concentration of 13.3 mcg/mL. The dose was escalated to 1250 mg q 12 hours resulting in a steady state serum trough concentration of 13.6 mcg/mL, and then to 1500 mg q12 hours, yielding a 9-hour concentration of 21.3 mcg/mL following the 4th 1500 mg dose, which was deemed therapeutic. Serum creatinine values ranged from 0.80 to 1.02 throughout the course of vancomycin therapy. The patient improved clinically, and blood cultures became sterile after three days of vancomycin. On day 6 of vancomycin the patient developed pruritus and a palpable nonblanching erythematous rash on her legs and buttocks (Figure 1). On the following day, the appearance of the rash was worse. Laboratory evaluation included complement levels, HIV antibody test, HCV PCR, antinuclear cytoplasmic antibody, antinuclear antibodies, serum cryoglobulin, and urinalysis, which were all negative/within normal range. Transesophageal echocardiography did not reveal any valvular vegetations. The dermatologist was consulted and performed a skin biopsy which showed findings consistent with leukocytoclastic vasculitis (Figure 2). Subsequently, we replaced vancomycin with linezolid. The other medications that the patient was on were thought to be a much less likely cause of the rash and were maintained. In the following days, without adding steroids or other treatments, the rash improved spontaneously. Complete recovery was noted 18 days thereafter.

Leukocytoclastic vasculitis (LV) is a small vessel inflammatory disease that is limited to the skin, predominantly of the lower extremities, and usually spares palms and soles. The most common skin manifestation is palpable purpura. Other skin manifestations include maculopapular rash, bullae, papules, nodules, ulcers, and livedo reticularis. There is no specific laboratory test for LV. The diagnosis is based on the clinical picture and histopathologic features of the skin biopsy. It is thought that the pathogenesis involves circulating immune complexes being deposited into vessel walls and activating the complement pathway. Causes of LV include drugs, infection, connective tissue disease, and malignancy. Drugs may act as haptens and activate the immune response. An estimated 10–20% of all cutaneous vasculitis cases are attributed to drug administration including *β*-lactams, diuretics, NSAIDs, methotrexate, azathioprine, etanercept, cyclosporine, allopurinol, sulfasalazine, gold salts, antithyroid agents, anticonvulsants, and antiarrhythmics [1]. By far, antibiotics have been the most common drugs reported to cause cutaneous vasculitis, especially *β*-lactams [2]. Vancomycin is well recognized as the cause of different types of hypersensitivity reactions, including red man syndrome, IgE-mediated anaphylaxis, and immune-mediated skin reactions such as vancomycin-related linear IgA bullous dermatosis [3–5]. 

Vancomycin-related leukocytoclastic vasculitis has rarely been reported [6–9]. From previous published case reports, vancomycin-associated small vessel vasculitis can develop after only a single dose of vancomycin [6]. The onset of vasculitis can occur as late as one month after administration [7, 8]. In one report LV developed after completing a 21-day treatment course and cessation of vancomycin for six days [9]. The time to recovery varies between days and weeks. Most cases of LV are self-limited and resolve without sequelae. The preferred treatment is withdrawal of the suspected offending agent(s) [7]. Oral corticosteroids have been used for LV; however, their efficacy is not impressive. Most of the patients had no change in disease course and one-third showed transient improvement, but relapse was observed when corticosteroids were tapered [10]. Colchicine and dapsone may be used in patients with limited palpable purpura without ulceration. 

We were not able to identify alternative causes for the vasculitis in our case, and the time course was consistent with vancomycin-associated LV. In the presence of newly developed palpable purpura, vancomycin has to be considered as a potential cause of leukocytoclastic vasculitis, and discontinuing the offending medication is the most effective approach.
